# N-acetylcysteine improves antitumoural response of Interferon alpha by NF-kB downregulation in liver cancer cells

**DOI:** 10.1186/1476-5926-11-4

**Published:** 2012-12-04

**Authors:** Nelson Alexandre Kretzmann, Eduardo Chiela, Ursula Matte, Norma Marroni, Claudio Augusto Marroni

**Affiliations:** 1 Post-Graduation Program in Medicine: Hepatology. Universidade Federal de Ciências da Saúde de Porto Alegre. Brazil. Universidade Federal de Ciências da Saúde de Porto Alegre, Porto Alegre, RS, CEP: 90050-170, Brazil; 2 Hospital de Clínicas de Porto Alegre, Porto Alegre, RS, CEP: 90035-903, Brazil; 3 Pos-Graduated Program in Cell Biology, Universidade Federal do Rio Grande do Sul, Brazil, Porto Alegre, RS, CEP 91501-970, Brazil

**Keywords:** N-acetylcysteine, Interferon alpha, NF-kB, Liver cancer cells

## Abstract

**Background:**

Liver cancer is one of the most common malignancies in the world and at the moment, there is no drug intervention effective for the treatment of liver tumours. Investigate the effect of N-acetylcysteine (NAC), which has been studied for its antitumoural properties, on the toxicity of hepatocarcinoma (HCC) cells *in vitro* when used with the drug interferon alpha-2A (IFN), which is used clinically to treat HCC.

**Results:**

NAC, IFN and NAC plus IFN reduced cell viability, as determined by MTT assay. More importantly, NAC potentiates the cytotoxic effect of IFN, with the best response achieved with 10 mM of NAC and 2.5 x 10^4^ of IFN. These results were confirmed by Annexin/PI staining through flow cytometry and morphologic analyses. Co-treatment reduced the expression of the nuclear transcription factor kappa-B (NF-kB). In a similar way to NAC, RNAi against p65 potentiated the toxic effect of IFN, suggesting that, indeed, NAC may be enhancing the effect of IFN through inhibition of NF-kB.

**Conclusions:**

Our results support the notion that NAC may be an important drug for the treatment of liver tumours as primary or adjuvant therapy. IFN has a limited clinical response, and therefore, the anti-proliferative properties of NAC in the liver should be explored further as an alternative for non-responders to IFN treatment.

## Background

Hepatocarcinoma (HCC) is the most common primary malignancy of the liver, typically observed as a complication of chronic liver disease. It is the fifth most common tumour worldwide, with more than 700,000 new cases per year [1]. Cirrhosis of different etiologies such as alcohol, primary biliary cirrhosis, or chronic infection with hepatitis B or C (HBV, HCV) are risk factors that predispose patients to HCC [2]. The development of HCC is a complex process, with the accumulation of genetic and epigenetic alterations, which pass through the events of tumour initiation, promotion and progression [2-4].

HCV chronic infection can induce chaotic cellular signalling, raising tumour cells with activation of epidermal growth factor (EGF) [5] and NF-kB, contributing to tumour development and survival of infected cells [6]. Interferon (IFN) is the most used drug in chronic hepatitis and HCC due to its properties of immune response activation and also regulation of differentiation and cell growth. IFN has also shown satisfactory results mainly in treating hematologic malignancies and Kaposi’s Sarcoma, among other diseases [7]. In HCC, studies have shown that IFN does not decrease metastasis or recurrence [8].

Other studies have shown that the progression of HCC is accompanied by activation of nuclear factor-kappa B (NF-kB) [6,9]. NF-kB is a transcription factor that plays an important and decisive role both in normal situations and in the coordination of adaptive immune responses, regulating the expression of many cellular mediators [10]. The family of NF-kB/Rel comprises five subunits, called p50, p52, p65 (RelA), c-Rel, and RelB. The activation of NF-kB requires the phosphorylation of its physiological inhibitors (particularly IkBα) in serine specific sites (Ser-32 and Ser-36), a process mediated by the complex inhibitor kappa kinases (IKKs) [11]. One of the documented functions of NF-kB is its ability to promote cellular survival due to induction of specific genes that inhibit apoptotic machinery in both normal and malignant cells [11,12]. NF-kB also prevents necrosis by inducing genes encoding antioxidant proteins [12-14]. Since NF-kB is a usual pathway that promotes resistance to drugs and radiation by tumoural cells, inhibition of NF-kB seems to be promising in improving the efficacy of conventional anti-cancer therapies [15,16].

NF-kB is also directly involved in oxidative stress and inflammation [12,17]. *N*-acetylcysteine (NAC) is one of the most used antioxidant drugs in liver diseases [18,19] and is known to be able to increase the levels of glutathione and also act as a free radical scavenger. Cell culture and animal studies have shown that NAC can protect normal cells, but not malignant cells, from the toxic effects of radiotherapy and chemotherapy [20]. The administration of NAC may have a role in cancer prevention and even in the treatment of some forms of cancer, as DNA induced damage can be completely blocked by NAC [21,22]. We herein tested the antitumoural effect of NAC on HCC cells and its relationship with the NF-kB pathway.

## Methods

### Cell culture and treatment

Human HepG2 and Huh7 HCC cells were obtained from the American Type Culture Collection (ATCC, Manassas, VA, USA). Stock cells were routinely grown as monolayer cultures in Dulbecco’s Modified Eagle’s Medium (DMEM) supplemented with 10% foetal bovine serum, penicillin (100 U/mL), streptomycin (100 mg/mL), glutamine (4 mM), and pyruvate (100 mg/mL) in a humidified 5% CO_2_ atmosphere at 37°C and the medium was changed every other day. Cells were maintained in T75 culture flasks and subcultured once a week in a total volume of 10 mL of complete medium. Cell culture reagents were purchased from Gibco (Invitrogen, Carlsbad, CA, USA), and culture flasks and dishes were purchased from TPP (Techno Plastic Products, Switzerland).

Twenty-four hours before treatments, 10^5^ HepG2 and Huh7 cells were replated in 6-well plates containing IFN-α 2A (Blausiegel Ind Ltda, SP-Brazil) at concentrations ranging from 0 to 10^5^ IU/mL and NAC (Sigma, Brazil) at final concentrations of 5, 10 and 20 mM. Both drugs were first diluted in PBS and then in DMEM to the final concentrations. Commercial p65 siRNA (250 mM) (Cell Signaling Biotechnology, Danvers, MA, USA) was used to suppress the NF-kB pathway, as described below. Cells were harvested after 24, 48, 72 and 96 h of treatment. Untreated cells used as controls (CO) were incubated in standard conditions. All experiments were performed in triplicate.

### Cell viability assay

For the cell viability assay 3-(4,5-dimethyl-thiazol-2-yl)-2,5-diphenyl-tetrazolium bromide (MTT, Sigma, Brazil) and confluent HepG2 and Huh7 cells were replated in 96-well culture plates, at a density of 3 x 10^3^ cells/well, in a total volume of 200 μL of complete medium.

The MTT assay was carried out as described by Denizot and Lang [23]. Briefly, after exposure of cells to IFN-α, NAC, NAC plus IFN-α, or siRNA (p65 or control) culture media was changed to serum-free media containing dissolved MTT (5 mg/mL). After 4 h, serum-free culture media containing MTT was discarded and DMSO was added to each well to dissolve the precipitate. The optical density was measured at 492 nm using a microtiter plate reader (Zenyth 200rt Microplate Reader; Anthos, Austria).

### Apoptosis analysis: Flow Cytometry and Fluorescent microscopy

Apoptosis was assessed using annexin-V conjugated with FITC (fluorescein isothiocyanate). HepG2 and Huh7 were treated with IFN-α, NAC or NAC plus IFN-α for 24, 48 or 72 h, as indicated. After treatment, cells were washed twice with PBS, and stained with PI and FITC-annexin–V (Apoptosis & Necrosis Quantification Kit, Biotium Hayward; CA USA) for 15 min in the dark. Cells were immediately analysed on GUAVA flow cytometer for PI and FITC-annexin–V staining.

Apoptosis was also evaluated by examining Annexin–V FITC and PI staining under fluorescent microscopy. Briefly, HepG2 and Huh7 cells were replated in 96-well culture plates, at a density of 3 x 10^3^ cells/well. Then cells were treated with IFN-α, NAC or NAC plus IFN-α for 48 or 72 h. After treatment, cells were washed twice with PBS and stained with PI and annexin–V FITC (Apoptosis & Necrosis Quantification Kit, Biotium Hayward; CA USA) for 15 min in the dark. Cells were immediately analysed using the Olympus FluoView™ 1000 microscope (CME-UFRGS).

### Western Blot Analysis

For western blot analysis of p65 expression, cell homogenates were prepared in 0.25 mM sucrose, 1 mM EDTA, 10 mM Tris and 1% protease inhibitor cocktail. The mixture was incubated for 30 min at 4°C and centrifuged for 30 min at 1,3000×g at 4°C. The supernatants were kept to analyse cell extracts. Samples containing 15 ug of protein were separated by sodium dodecyl sulphate-polyacrylamide gel electrophoresis (9% acrylamide) and transferred to a nitrocellulose membrane. Non-specific binding was blocked by preincubation in PBS containing 5% bovine serum albumin for 1 h. Membranes were then incubated overnight at 4°C with polyclonal anti-p65 (65 kDa) (Cell Signaling Technology, Danvers, MA) and anti-β-actin (42 kDa) (Sigma Brazil), prepared as described by Guitierrez [24]. Bound primary antibody was detected by incubation with HRP-conjugated anti-rabbit antibody for 2 h (DAKO, Glostrup, Denmark) and bands were revealed using an enhanced chemiluminescence detection system (ECL kit, (GE Healthcare, Piscataway, NJ, USA). The densities of the specific bands were quantified with an imaging densitometer (Scion Image, Maryland, MA) [25].

### Silencing of p65 expression with siRNA

Briefly, HepG2 and Huh7 cells were replated in 12-well plates at 10^4^ cells/well 24 hours after culture media was changed to serum-free media. Cells were then washed twice with PBS before transfection. Specific predesigned small interfering RNAs (siRNA) to p65 were ordered from Cell Signaling Biotechnology (CA, USA) and transfected into HepG2 and Huh7 cells with the accompanying transfection reagent according to the manufacturer’s instructions. siRNA with equivalent %GC nucleotide content and FITC labelling was used as a control. Cells were assayed 24 h after siRNA duplex transfection. The effect of p65 suppression was monitored by p65 mRNA levels.

### RNA isolation and Real-Time PCR

Total RNA from cells subjected to different treatments was extracted using the RNeasy Mini Kit (Qiagen, Germany). RNA was quantified and the quality tested by photometric measurement on a Nanodrop apparatus (Wilmington, DE, USA). Only highly purified RNA (A260/A280>1.95) was used. cDNA synthesis was performed using the SuperScript™ III/RNaseOUT™ Enzyme Mix 2 and 50 μM oligo(dT) random primers (Invitrogen, Carlsbad, CA, USA). The cDNA was stored at −20°C.

Oligonucleotide primers for the amplification were obtained from the Harvard Medical School Primer Bank ( http://pga.mgh.harvard.edu/primerbank/). The primer sequences used were as follows: *p65* Forward Primer 5^′^-TTGAGGTGTATTTCACGGGACC-3^′^ and Reverse Primer 5^′^-GCACATCAGCTTGCGAAAAGG-3^′^, and *GAPDH* Forward Primer 5^′^-CCCATCACCATCTTCCAGG-3^′^ and Reverse Primer 5^′^-GAGATGATGACCCTTTTGGC-3^′^). PCRs were carried out in a final volume of 25 μl, containing 1 μM of both primers, 1x SYBR Green Supermix (Applied Biosystems), and variable amounts of cDNA templates. The program profile used for *p65* amplification was the following: 95°C for 2 min, 45 cycles of denaturation for 30 sec at 95°C, annealing for 15 sec at 52°C and extension for 30 sec at 60°C. The program profile used for GAPDH was 95°C for 2 min followed by 45 cycles of denaturation, annealing and extension for 30 sec each at 95°C, 65°C and 60°C, respectively [26,27]. Thermal cycling was performed in a Mx3000P™ real-time PCR system Stratagene Thermocycler (GE, USA). Data were analysed with the accompanying software MX PRO System Software, using 2^ΔΔCt^ formula.

### Statistical analysis

Means and standard errors of the mean (SEM) were calculated. Significant differences between means were evaluated by analyses of variance and in the case of significance; a Newman–Keul’s post-hoc test was also applied. Real-time PCR data was analysed by a Student’s t-test. A difference was considered significant when P was less than 0.05. SPSS+ version 13.0 statistical software was used.

## Results

### NAC and IFN-a decrease cell viability of liver cancer cells

**Figure 1:**
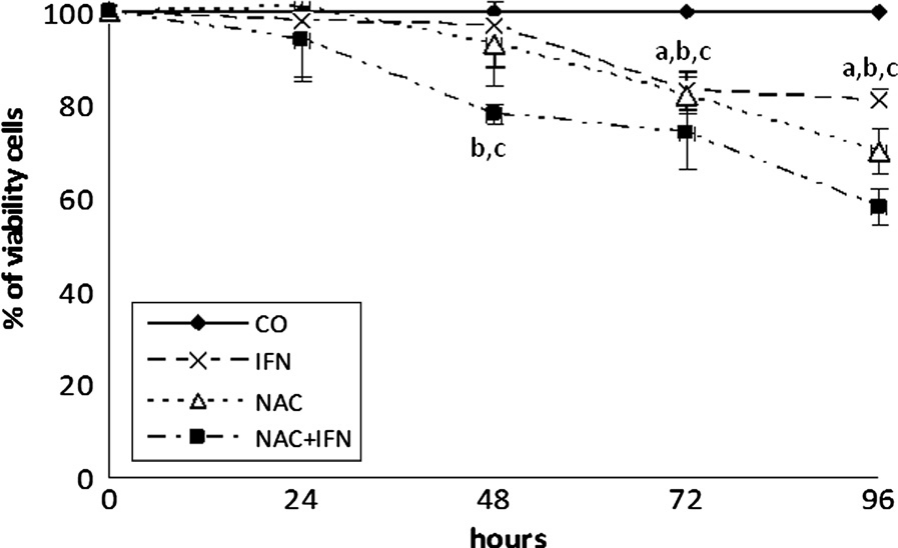
**NAC potentiates the effect of IFN by decreasing cell viability of HCC HepG2 cell line.** Treatment with IFN or NAC, at 2.5x10^4^ U/mL and 10 mM, respectively, significantly reduced cell viability after 48, 72, and 96 h of treatment. Treatment with NAC+IFN in the same doses significantly reduced cell viability after 24, 48, 72, and 96 h of treatment. Values are shown as means and standard errors of the mean (SEM). a-IFN x CO p<0.05. b- NAC x CO p<0.01. c- NAC+IFN x IFN p<0.05.

**Figure 2:**
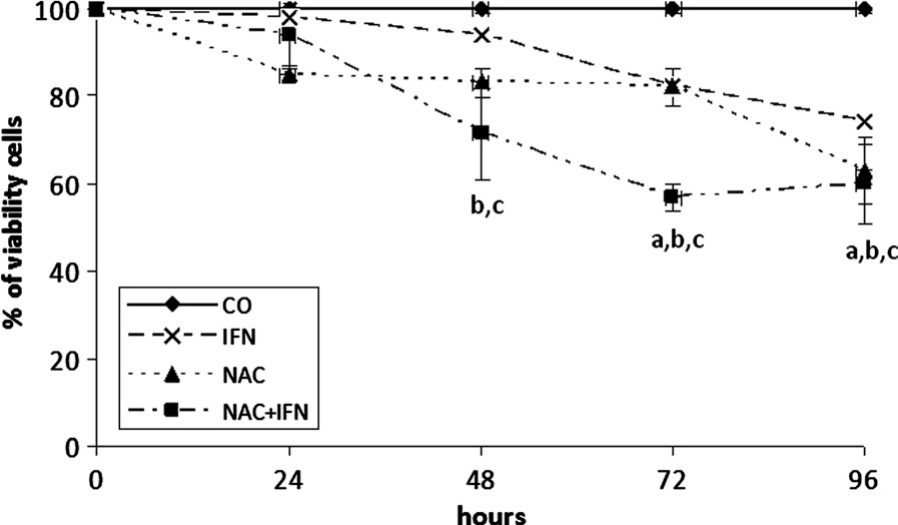
**NAC potentiates the effect of IFN by decreasing cell viability of HCC Huh7 cell line.** Treatment with IFN or NAC, at 2.5x10^4^ U/mL and 10 mM, respectively, significantly reduced cell viability after 48, 72, and 96 h of treatment. Treatment with NAC+IFN in the same doses significantly reduced cell viability after 24, 48, 72, and 96 h of treatment. Values are shown as means and standard errors of the mean (SEM). a-IFN x CO p<0.05. b- NAC x CO p<0.01. c- NAC+IFN x IFN p<0.05.

The ideal doses of IFN-α (2.5 x 10^4^) and NAC (10 mM) were found through dose curves using concentrations ranging from 0 to 10^5^ IU/mL for IFN-α, and 5 to 20 mM for NAC (data not shown). Both drugs had a dose-dependent effect. IFN-α at a concentration of 2.5 x 10^4^ U/mL (96 hours) decreased cell viability to about 30% in HepG2 and Huh7 cells, while 10 mM NAC reduced cell viability in both cell lines at 48, 72, and 96 hours. NAC and IFN-α combined have a more potent and earlier effect than any of these drugs alone, reducing cell viability to around 50% at 96 hours (Figures 1 and 2).

### Inhibition of NF-kB pathway by NAC induces apoptosis in HCC cells

**Figure 3:**
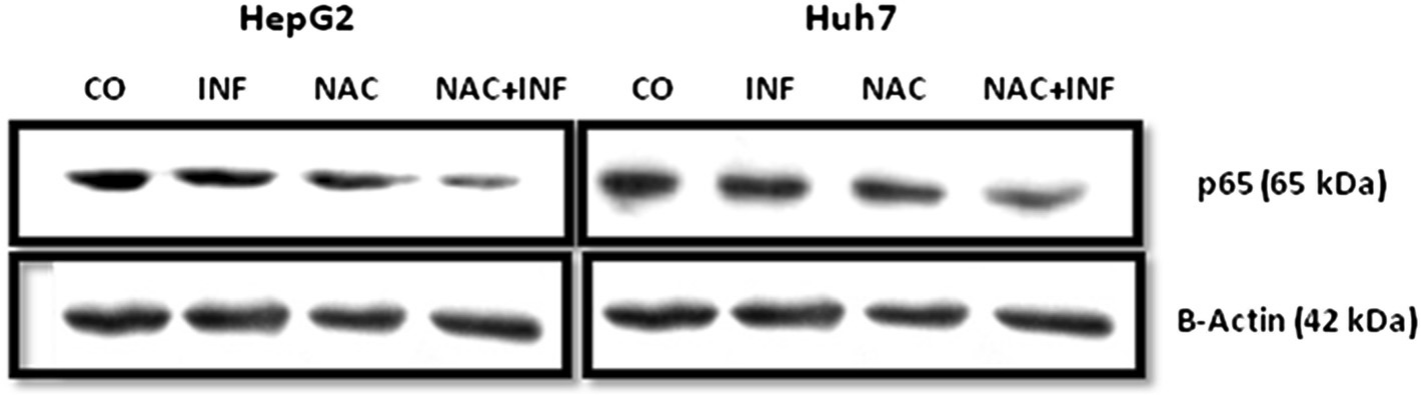
**NAC and IFN synergistically inhibit p65 expression in HepG2 and Huh7 cells.** Immunoblotting analysis of p65 subunit and β-actin of cells treated for 72 h with IFN 2.5x10^4^ U/mL and/or NAC 10 mM.

**Figure 4:**
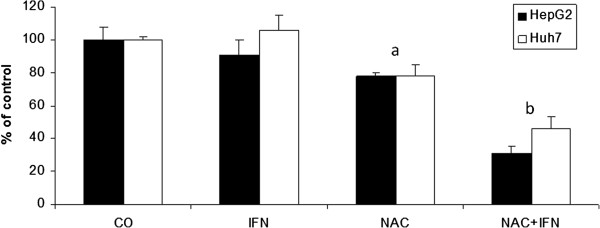
**NAC and IFN synergistically inhibit p65 expression in HepG2 and Huh7 cells.** Quantification of band density with an imaging densitometer. Results are representative of three independent experiments. Values are shown as means and standard errors of the mean (SEM).a- NAC x CO p<0.01. b- NAC+IFN x CO x IFN x NAC p<0.01.

To test the role of NAC in the NF-kB pathway and induction of apoptosis, we analysed cells by flow cytometry and fluorescent microscopy to detect annexin V, and by western blot to detect NF-kB p65 subunit expression. NAC alone decreased the NF-kB p65 subunit expression in HepG2 and Huh7 cells and, more importantly, co-treatment with NAC plus IFN-α synergistically reduced the NF-kB p65 subunit expression after 72-hour treatment (Figures 3 and 4).

**Figure 5:**
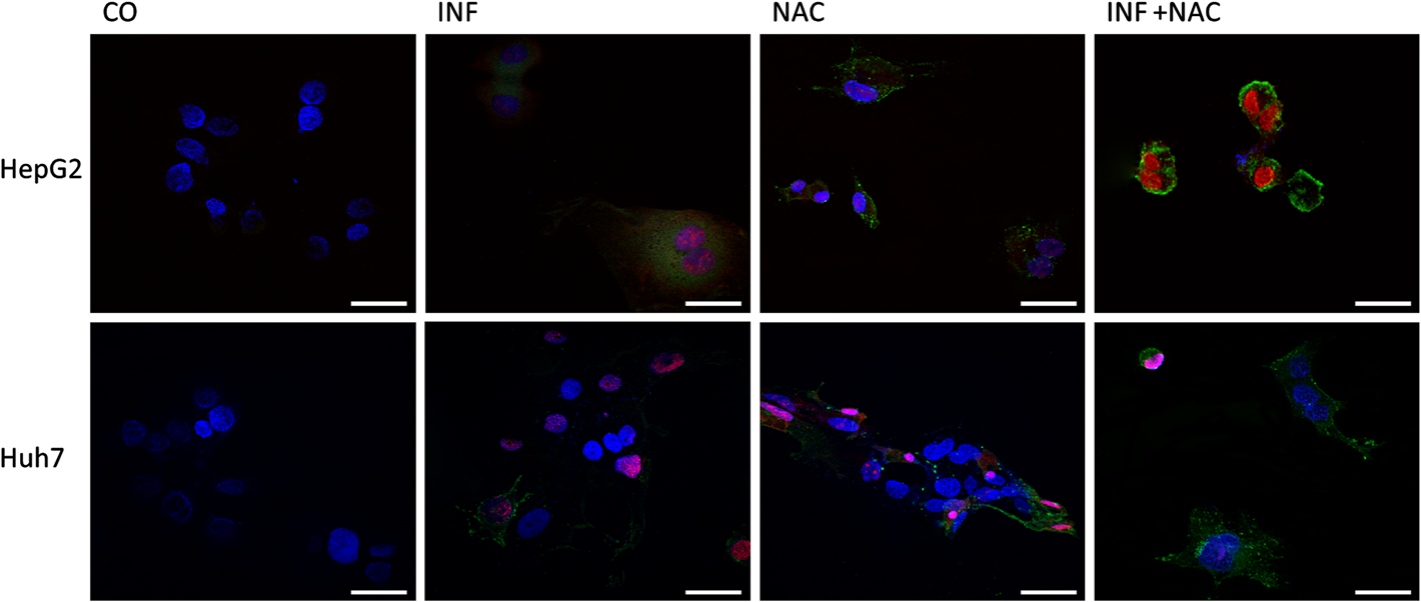
**NAC and IFN treatment induce apoptosis in HCC cells.** Cells were treated with IFN 2.5x10^4^ U/mL and/or NAC 10 mM for the indicated time periods. Fluorescence microscopy of HepG2 and Huh7 cells stained with annexin and PI. FITC-labelled Annexin V show apoptotic cells (green) and PI stained cells (red) indicate necrosis or late apoptosis. DAPI stained nuclei (blue). Magnification used was 60x. Bar, 25μm.

**Figure 6:**
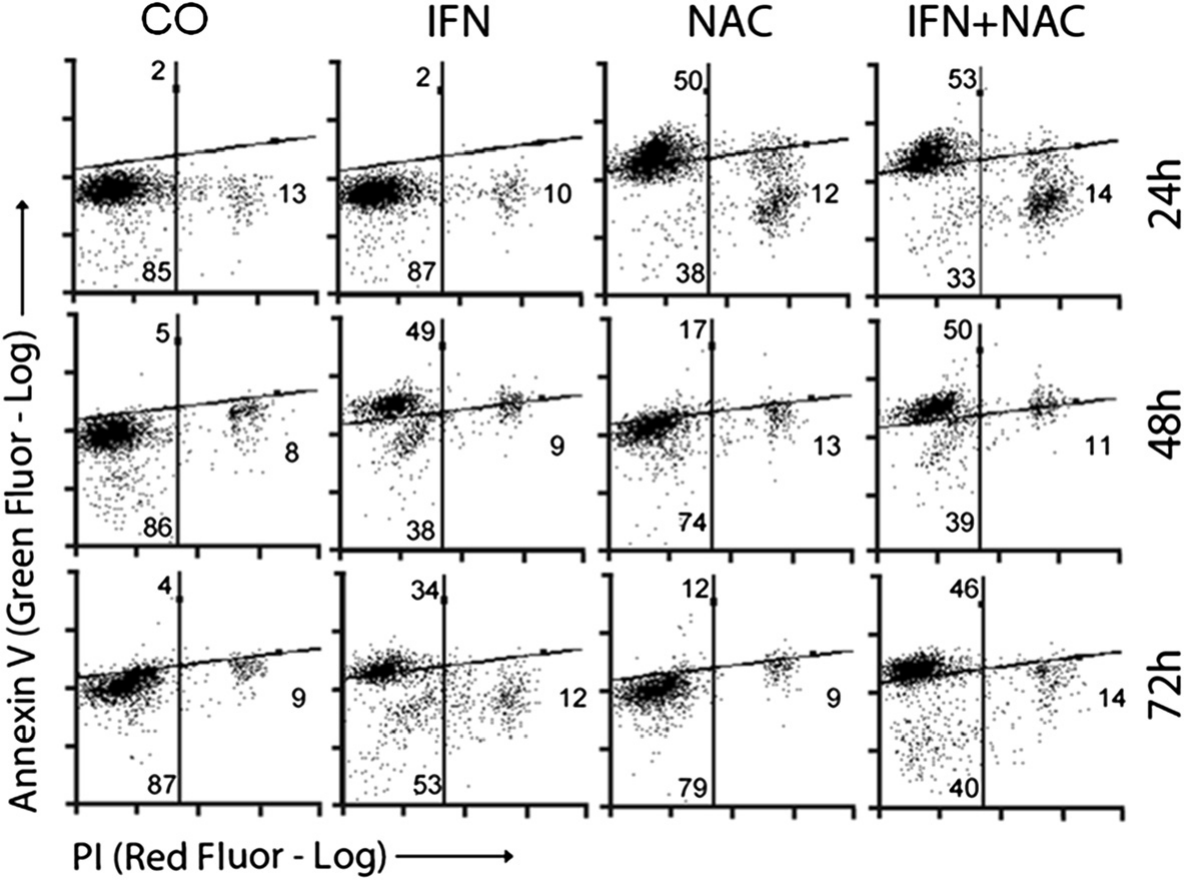
**Quantification of marked cells was done by flow cytometry of HepG2 cells.** Annexin V staining (Green Fluor-Log-Y) and PI staining (Red Fluor-Log-X) of HepG2 (**B**) and Huh7 (**C**) cells are shown. Values are shown on quadrants as means and standard errors of the mean SEM).

**Figure 7:**
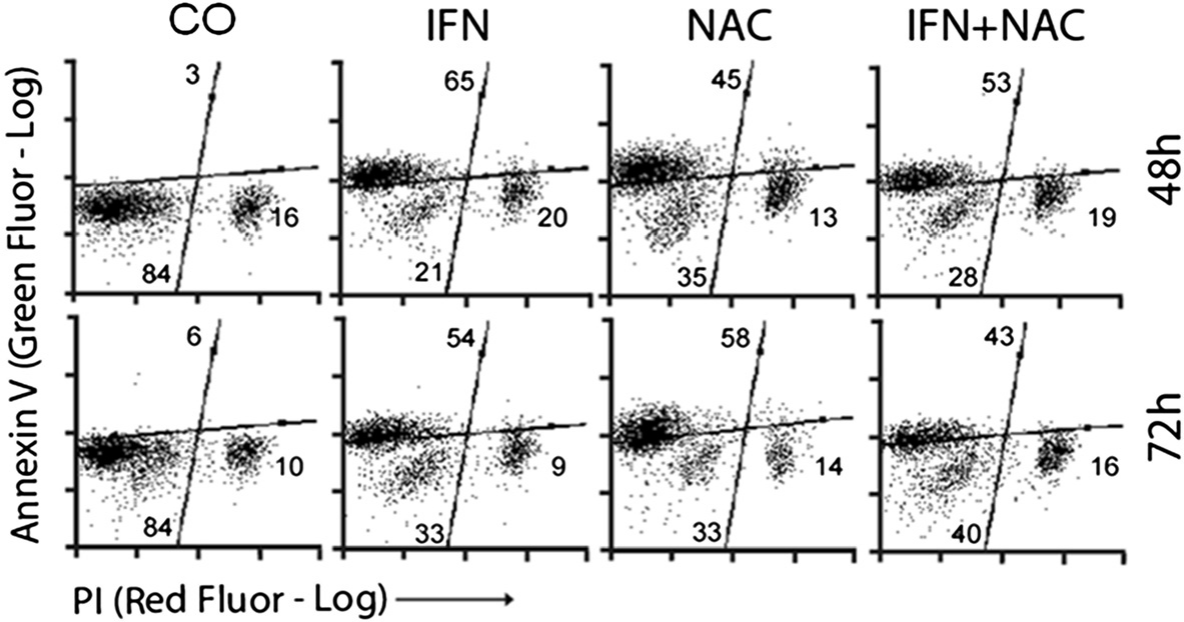
**Quantification of marked cells was done by flow cytometry of Huh7 cells.** Annexin V staining (Green Fluor-Log-Y) and PI staining (Red Fluor-Log-X) of HepG2 (**B**) and Huh7 (**C**) cells are shown. Values are shown on quadrants as means and standard errors of the mean SEM).

On annexin V/PI analysis through fluorescence microscopy and flow cytometry, both NAC and IFN-α seemed to have proapoptotic effects in both cell lines (Figures 5, 6 and 7). Interestingly, cells presented a different profile of sensitivity to treatments. HepG2 cells were more sensitive to treatment with NAC, presenting positive annexin-V staining at 24 h of treatment, while Huh7 cells were more sensitive to IFN. NAC potentiated the proapoptotic effect of IFN mainly in HepG2 cells, in which the reduction in NF-kB expression was also higher with co-treatment (Figures 3 and 4).

### NAC increases IFN-a antitumoural responses mediated by NF-kB Pathway inhibition

**Figure 8:**
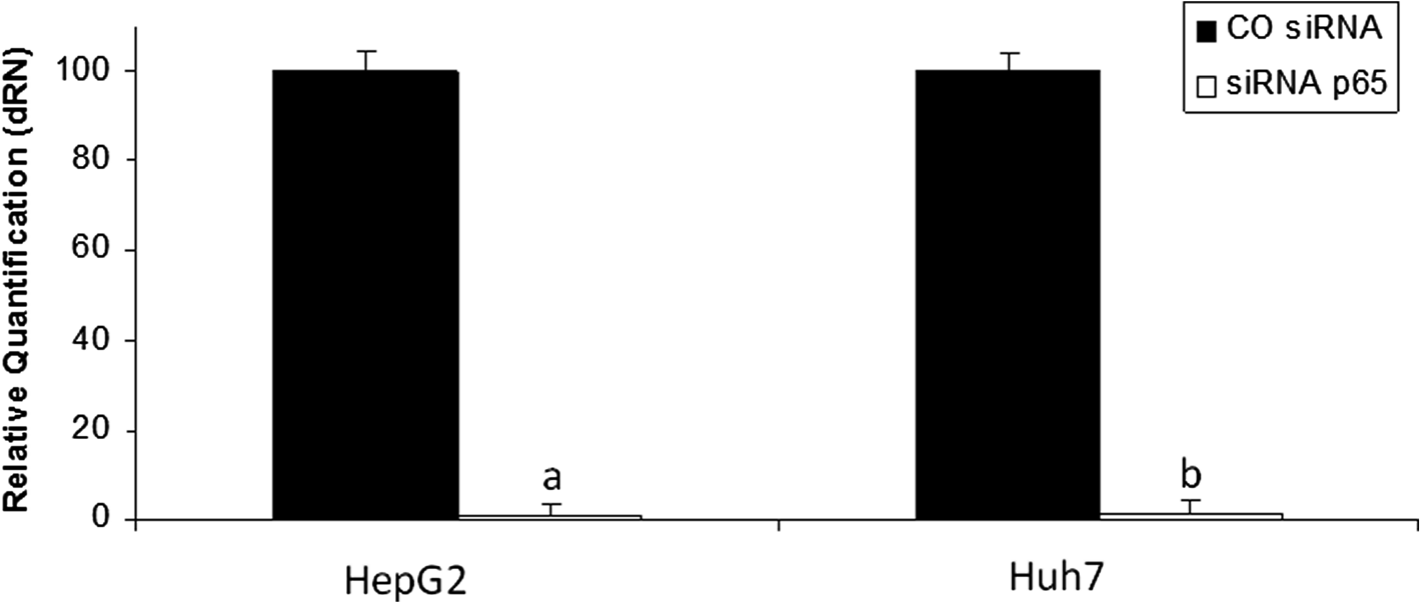
**Knock down of p65 subunit shown by real-time PCR.** Relative quantification of p65 normalised by the expression of GAPDH in HepG2 and Huh7 cells 24 hours after transfection. Values are shown as means and standard errors of the mean (SEM). a- siRNAp65x COsiRNA p<0.01-HepG2. b- siRNAp65x COsiRNA p<0.01-Huh7.

We then explored the role of the NF-kB pathway on NAC and IFN-α toxicity using siRNA-mediated p65 knockdown (KD cells). At 24 h post-transfection, a greater reduction of 95% of p65 expression levels was observed both through fluorescence microscopy (data not shown) and real-time PCR (Figure 8).

**Figure 9:**
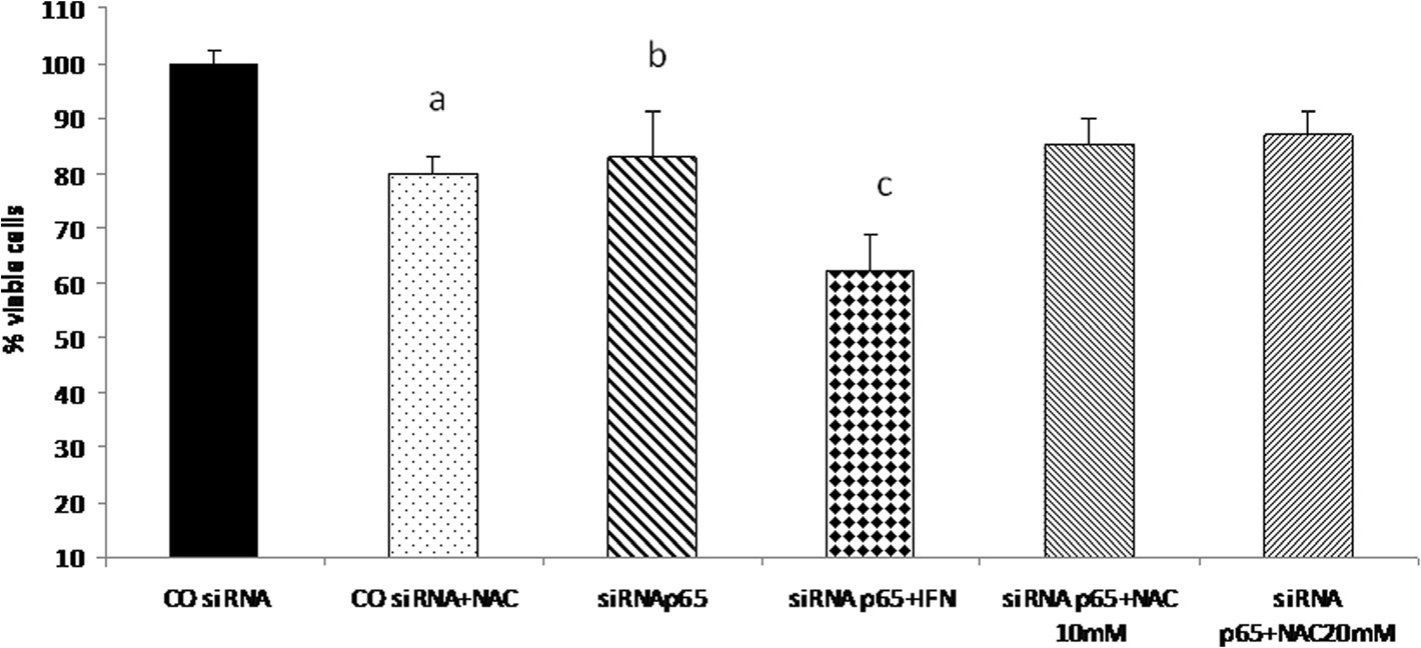
**Effects of IFN and NAC on cell viability of HepG2 cells with p65 knock down.** HepG2 cells were treated 24 h after siRNA duplexes transfection with IFN 2.5x10^4^ U/mL and/or NAC 10 mM, and cell viability was determined after 24 hours of treatment. Values are shown as means and standard error of media (SEM). a- COsiRNA+NAC x COsiRNA x siRNAp65 p<0.01. b- siRNAp65 x COsiRNA x siRNAp65+IFN p<0.05. c- siRNAp65+IFN x COsiRNA x COsiRNA +NAC x siRNAp65 x siRNAp65+NAC (10 and 20 mM) p<0.05.

**Figure 10:**
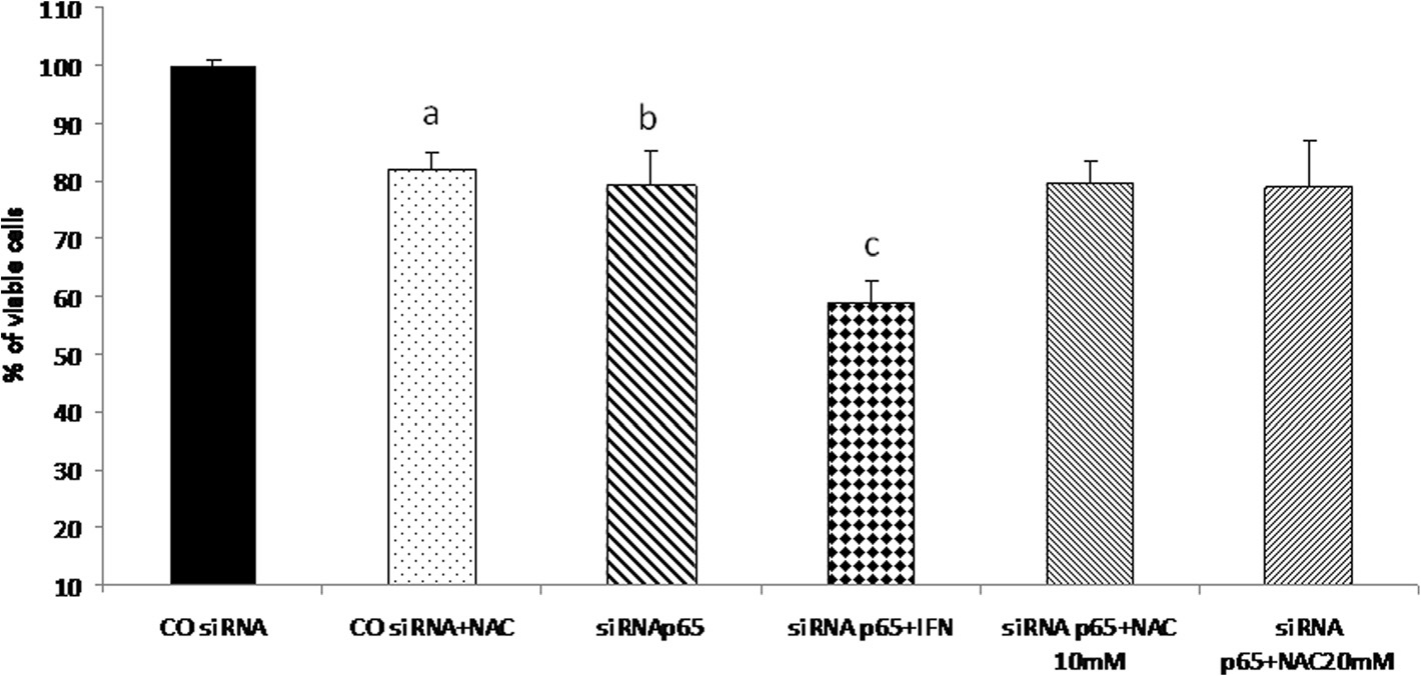
**Effects of IFN and NAC on cell viability of Huh7 cells with p65 knock down.** Huh7 cells were treated 24 h after siRNA duplexes transfection with IFN 2.5x10^4^ U/mL and/or NAC 10 mM, and cell viability was determined after 24 hours of treatment. Values are shown as means and standard error of media (SEM). a- COsiRNA+NAC x COsiRNA x siRNAp65 p<0.01. b- siRNAp65 x COsiRNA x siRNAp65+IFN p<0.05. c- siRNAp65+IFN x COsiRNA x COsiRNA +NAC x siRNAp65 x siRNAp65+NAC (10 and 20 mM) p<0.05.

The combined treatment with p65 siRNA with IFN-α for 24 h showed a decrease in cell viability that was comparable to that observed in NAC plus IFN-α treatment. On the other hand, suppression of p65 did not sensitise cells to NAC, suggesting that the mechanism of action of NAC primarily involves reduction of NF-kB (Figures 9 and 10).

## Discussion

Given that the efficiency of IFN-α is only marginal in treating HCC, our study aimed to evaluate the effect of NAC on IFN-α toxicity, and how the co-treatment of NAC and IFN-α modulates cell death and growth inhibition in HCC human cell lines. We showed that NAC decreased cell viability through downregulation of the NF-kB pathway and induction of apoptosis. More importantly, NAC increased the toxicity of IFN-α through an additive induction of apoptosis and a synergistic decrease of NF-kB expression in HCC cells, pointing to different targets being modulated by IFN-α and NAC.

IFN-α has been shown to reduce the incidence of pre-neoplastic foci and cancer in liver cancer models [28,29]. Our results *in vitro* using 2.5 x 10^4^ U/mL showed a decrease in cell viability of around 30%, which could be considered a poor response. These results are in agreement with the poor response observed clinically, in which only around 30% of the patients respond to treatment [30]**.** These data confirmed that development of alternative compounds to treat HCC, such as NAC tested here, is necessary.

The selective induction of apoptosis in cancer cells is an exciting possibility for the selective development of future therapies to treat HCC [31-33]. Knowing that one of the IFN-α mechanisms of action involves apoptosis through p53 induction and the activation of caspases [34-36], here we used cell lines with a different p53 status in order to establish the mechanisms involved in the toxicity of IFN-α and NAC in HCC cells. Some studies indicated that the presence of p53 would facilitate apoptosis induction [22,37]. In our study we demonstrated that, despite leading to apoptosis in a p53-independent way, NAC triggered apoptosis in HepG2 p53 functional cells after 24 h of treatment, while in p53-deficient cells (Huh7) this effect was observed only after 48 hours of treatment. Furthermore, in HepG2 cells, NAC not only potentiated the effect of IFN-α in reducing cell viability, but also increased labelling with annexin V after 24 h without increasing the overall amount of apoptosis. More interestingly, after 48 h and 72 h of treatment with NAC, we did not observe any more annexin-positive cells in the HepG2 cells, while in IFN-α and NAC plus IFN-α treatments, we still observed annexin-positive cells after 48 h and 72 h. This suggests that NAC triggered apoptosis in some of the HepG2 cells, and those that remained were resistant to treatment, while co-treatment surpassed this resistance. This finding is an important point to be considered in clinical approaches using NAC or co-treatment with IFN-α.

High expression of pro-angiogenic factors such as hypoxia-inducible factor-1α and cell growth/survival factors such as CD24 and activation of inflammatory signalling pathways such as Wnt/β-catenin, nuclear factor-kappa B and signal transducer and activator of transcription 3 predict early recurrence of HCC [4,38]. Wnt/B-Catenin signaling is one of many pathways that are also altered in HCC, but it is also known that it responds to both NAC and INF used alone. It is conceivable that the use of both drugs could also have a synergistic effect on this pathway as well [39-41]. p53 and other transcription factors have been closely linked to cancer and related therapies. Data suggest that IkB simultaneously downregulates NF-kB activation and sequesters p53 in the cytoplasm, thus enhancing NF-kB-regulated apoptosis but blocking p53-dependent apoptosis [42]. It is thought that several carcinogens and tumour promoters act through the constitutive activation of NF-kB [16,43], which induces the resistance of cancer cells to chemotherapeutic agents and radiation [44]. The balance between proliferation and cell death is a decisive factor in the progression or inhibition of carcinogenesis, and a variety of mechanisms can be activated or inactivated to induce apoptosis [33]. Antioxidant molecules that have a thiol group, such as NAC, have the ability to promote several of these mechanisms in different types of human tumours [13,45]. One of these mechanisms refers to upregulation of pro-apoptotic genes together with the downregulation of inhibitors of apoptosis genes, often accompanied by increased permeability of the mitochondrial membrane and release of cytochrome c, activating the caspase cascade. And all of these events are regulated by activation or inactivation of NF-kB [24,46,47]. Data from the present study confirm the findings of previous studies that showed a decrease in the expression of the p65 subunit using NAC or IFN-α [31,48-53]. More importantly, combined treatment further reduced levels of p65 in a synergistic way, again suggesting that NAC and IFN-α act in different pathways. Since several genes involved in the initiation, promotion and tumour progression are regulated by NF-kB and its activation suppresses apoptosis and promotes cell proliferation [16,54], the rational design of treatments that decrease NF-kB activity is a good strategy to treat malignancies, as observed here.

Confirming the involvement of NF-kB on the effect of NAC, we found that cells transfected with siRNA for the p65 (KD cells) had the same response of cells treated only with NAC. Furthermore, KD cells treated with IFN-α had the same response as the combined treatment with NAC plus IFN-α while knockdown of NF-kB did not alter the sensitivity to NAC. Altogether, these data suggest that the increase in growth inhibition shown by NAC is probably due to the inhibition of NF-kB pathway. Even though it has been shown that IFN-α may have a role in blocking the NF-kB activating pathway triggered by the hepatitis B virus [51], this was not observed in our experiments. IFN-α treatment alone showed only a slight decrease in NF-kB activation, suggesting that IFN-α may act through different mechanisms depending on cell type and context.

In conclusion, NAC potentiates the antitumoural effect of IFN-α, decreasing cell viability, increasing apoptosis and decreasing the expression of the p65 subunit of NF-kB. Considering that NF-kB is a molecule of great importance in the initiation, promotion and progression of tumours and that NAC acts on the inactivation of this pathway with a tumoural-specific toxicity, we suggest that NAC is a promising agent for use in HCC, primarily or as an adjuvant with IFN-α.

## Competing interest

The authors declare that they have no competing interests.

## Authors’ contributions

NAK made all experiments, data analysis and wrote the paper, EC had worked in cytometry analysis and results discuss, UM gave the laboratory supply and help in on the discussion of results and review the paper, NM gave the financial support and laboratory supply and CAM helped in article writing and revision of data. All Authors read and approved the final manuscript.
